# Impact of micro-environmental factors on survival, reproduction and distribution of *Oncomelania hupensis* snails

**DOI:** 10.1186/s40249-021-00826-3

**Published:** 2021-04-07

**Authors:** Mao-Mao Liu, Yun Feng, Kun Yang

**Affiliations:** 1 Nanjing Medical University, 101 Longmian Avenue, Nanjing, 211166 Jiangsu People’s Republic of China; 2 Jiangsu Institute of Parasitic Diseases, 117 Meiyuan Yangxiang, Wuxi, 214064 Jiangsu People’s Republic of China; 3 Key Laboratory of National Health and Family Planning Commission on Parasitic Disease Control and Prevention, Wuxi, People’s Republic of China; 4 Jiangsu Provincial Key Laboratory on Parasite and Vector Control Technology, Wuxi, People’s Republic of China

**Keywords:** *Oncomelania hupensis*, Micro-environment, Survival, Reproduction, Distribution, Impact

## Abstract

**Background:**

Schistosomiasis japonica is a chronic parasitic disease that seriously harms people's health. *Oncomelania hupensis* is the only intermediate host of *Schistosoma japonicum*. The micro-environmental factors surrounding the snail have a great impact on the survival, growth and reproduction of *O. hupensis*, but there are few relevant systematic analyses until the present. This scoping review aims to identify and summarize the micro-environmental factors that greatly affect *O. hupensis*, and to find gaps in research thus to provide directions for future in-depth studies.

**Main body:**

This scoping review searched databases with search terms of the combinations of “Micro(-)environment”, “*Oncomelania*” and their expanded aspects. A total of 133 original articles were recruited. Predefined data fields were extracted including research methods, influencing factors, and their effects on *O. hupensis*. Most studies focused on vegetation factors (54.1%), and other factors noted were soil composition (27.8%), water environmental factors (24.1%), and predator (3.0%), respectively. The factors with positive impacts included water level, pH value, soil temperature, soil humidity, the coverage and height of vegetation at suitable levels. This could provide more detailed information for *O. hupensis* habitat identification and prediction. The factors with negative impacts included plant extracts, snail control and disease prevention forests, and microorganisms with molluscicidal activities. It revealed a potential application as ecological molluscicides in the future. Factors such as physico-chemical properties of water, soil chemistry showed a gap in scientific studies, thus required further extensive research.

**Conclusions:**

Micro-environmental factors including water quality, soil composition as well as the technology and application of biomolluscicides (plant extracts and microorganisms) deserve more attention. Relative study findings on micro-environment have good potentials in snail control applications. Further studies should be implemented to investigate the impact of micro-environmental factors on snails and close the research gaps.

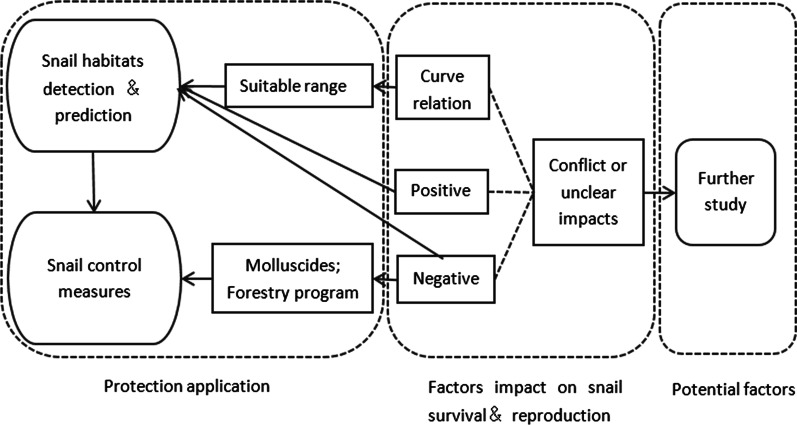

**Supplementary Information:**

The online version contains supplementary material available at 10.1186/s40249-021-00826-3.

## Background

Schistosomiasis japonica is a zoonotic disease caused by *Schistosoma japonicum,* which seriously harms people’s health, mainly lead to colon and liver lesions [1]. It is currently endemic in China, Indonesia and the Philippines [2]. In China, a total of 30 170 patients with advanced schistosomiasis japonica were reported in 2019 [3]. The prevention and treatment of schistosomiasis should not be ignored, including the control of the intermediate host snails.

*Oncomelania hupensis* is the only intermediate host of *S. japonicum* [4]. The distribution of *S. japonicum* infection is determined by the distribution of *O. hupensis* [5]. The control of *O. hupensis* can fundamentally reduce the infection rate of the population, which is essential for the prevention of schistosomiasis. Environmental factors have a great influence on the survival, growth and reproduction of snails [6–9]. Many researches focus on macro-environment factors, such as temperature, climate, rainfall. However, limited systematic analysis are available to investigate the effect of micro-environmental factors which is physically surrounding *O. hupensis*. Such micro-environmental factors include water quality, water level, soil, and vegetation. All are important to design an effective snail control strategy for the snail intermediate host [10, 11].

We hereby systematically reviewed the relevant micro-environmental factors with the following three purposes: First, we aimed to explore the impact of micro-environmental factors on the survival, reproduction and distribution of *O. hupensis*; second, we expected to identify the essential micro-environmental factors that greatly affect the snail distribution; and finally, we would like to find gaps in research thus provide directions for future in-depth studies.

## Methods

### Search strategy and selection criteria

We searched five Chinese and English databases [PubMed, EBSCOhost, Web of Science, China National Knowledge Infrastructure (https://www.cnki.net/), and Wanfang (http://www.wanfangdata.com.cn/index.html) database] with no restriction on time. The last retrieval time was December 2019. Search terms were the combinations of “Micro(-)environment”, “*Oncomelania*” and their expanded aspects including water environment, soil environment and vegetation, snail subspecies and other identified search terms (see Additional file 1).

**Fig. 1:**
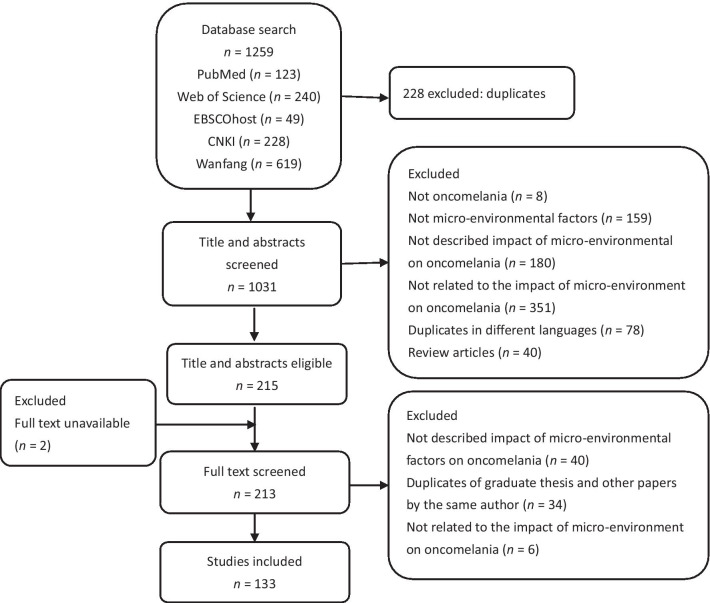
Flow chart of included articles

After removing duplicated articles, two authors screened the titles and abstracts of the articles according to the following inclusion criteria, respectively: (i) the article described impact of micro-environmental factors on the survival, reproduction and distribution of *O. hupensis*; (ii) only original studies were included. Articles without full text available were excluded. The exclusion criteria: (i) Not related to *Oncomelania* and micro-environment; (ii) not described or related to the impact of micro-environment on *Oncomelania*; (iii) duplicates in different languages; (iv) duplicates of graduate thesis and other papers by the same author; (v) review articles. The screening process was shown in Fig. 1.

### Data extraction

Two authors extracted the information from the recruited articles, respectively. According to the predefined data fields, the following information was extracted: research methods (experimental research/field investigation), influencing factors (water quality, water level, predator, soil physical and chemical properties, soil microorganisms, *O. hupensis* control and schistosomiasis prevention forest, plant extracts, plant microorganism, vegetation characteristics etc.), and effects on *O. hupensis* (positive/negative/curve/unknown). Positive effect means that the enhancement of micro-environmental factors was beneficial to the survival, reproduction or distribution of *O. hupensis*; negative effect means that the enhancement of micro-environmental factors is unfavorable to that; curve effect means that the relationship between micro-environmental factors and *O. hupensis* is not a simple linear relation, and there could be one or more suitable ranges.

## Results and discussion

A total of 1259 articles were retrieved by searching databases. After removing duplicates, and screening title/abstract/full-text, a total of 133 articles met the inclusion criteria (Fig. 1).

Among the relevant influencing factors of *O. hupensis*, water environmental factors, soil environment, vegetation and predator accounted for 24.1%, 27.8%, 54.1%, and 3.0%, respectively. Water level, water quality, soil temperature, humidity, some chemical indexes, vegetation height, coverage, plant extracts, snail control and schistosomiasis prevention forest, *Procambarus clarkia *(also known as crayfish) and *Ophiocephalus argus* (black carp) were mainly involved. Table 1 summarized the included articles and classified them according to relevant conclusions.

**Table 1:** Classification of included articles by micro-environmental factors covered

Micro-environmental factors (*n*, %)	Detailed factor	Effect	Description
Water environment (32^a^, 24.1%)			
Water level (25, 18.8%)	–	Curve relationship	An optimal range exists
Water quality (7, 5.3%)	Transparency (1)	Positive effects	*Oncomelania hupensis* were more likely to survive in water with high transparency
	Chemical properties (6)	Need further research	Results from literatures conflict
Soil environment (37^a^, 27.8%)			
Soil properties (31, 23.3%)	Soil humidity, temperature (18)	Curve relationship	An optimal range exists
	Soil pH value (7)	Curve relationship	An optimal range exists
	Soil fertility indexes (8^b^) organic compounds, total N, total P, total K, etc	Need further research	Results from literatures conflict
Soil microorganisms (6, 4.5%)	*Aspergillus fumigatus* SL-30, *Streptomyces violaceoruber*, *Xanthobacter autotrophicus,* etc	Negative effects	These microorganisms have molluscicidal activity
Vegetation (72^a^, 54.1%)			
Plant extracts (34, 25.6%)	Root, stem, and leaf extracts	Negative effects	These plant extracts have molluscicidal activity
Land cover vegetation characteristics (23, 17.3%)	Vegetation height, coverage (12)	Curve relationship	An optimal range exists
	Plant species (11) *Cynodon dactylon*, *Alternanthera philoxeroides*, *Pterocarya stenoptera* community and *Nerium indicum* community, *Liquidambar formosana*, *Sapium sebiferum*, etc	Negative effects	*O. hupensis* are less distributed in areas with these vegetation types
Snail control and schistosomiasis prevention forest (14, 10.5%)	–	Negative effects	The implementation of snail control and schistosomiasis prevention forest reduces the density of *O. hupensis*
Plant microorganisms (1, 0.8%)	Endophyte JJ18 of *Pseudolarix amabilis*	Negative effects	These microorganisms have molluscicidal activity
Predator (4^a^, 3.0%)			
*Procambarus clarkia, Ophiocephalus argus* (4, 3.0%)		Negative effects	Predation reduces the density of *O. hupensis*

In the second column, the numbers in brackets represent the number of articles on related factors

^a^Some of the articles discussed more than one influencing factors

^b^Two articles specified the subspecies *O. hupensis quadrasi* and showed that organic compounds had a positive impact on the snail survival

**Fig. 2:**
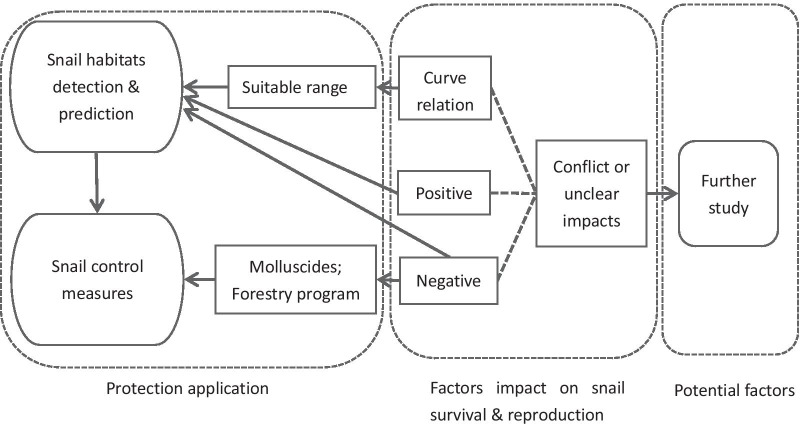
Application of micro-environment factors on *Oncomelania hupensis*

As shown in Fig. 2, micro-environmental factors that were beneficial to the growth of *O. hupensis* within a suitable range could be applied to the identification and prediction of the habitats of snails. Factors that negatively affect *O. hupensis* can be used in *O. hupensis* forecast and control. Factors that were less researched or showed contradictory results, leading to no definite conclusions at present, require more in-depth research.

## Micro-environmental factors with snail habitats prediction potentials

### Micro-environmental factors with curve relationship to *O. hupensis* survival

Factors like water level, pH value, soil temperature and humidity, vegetation coverage and height have a curve relationship with the survival of *O. hupensis*. There were suitable ranges of the factor indicators, beyond or below which was not conducive to *O. hupensis*. Water level is an important factor affecting *O. hupensis*, which has been widely studied. After the operation of the Three Gorges Dam on Yangtze River in China, the flooding time was shortened and the water level was lowered relatively, which was not conducive to the survival of *O. hupensis* [12, 13]. Field and experimental studies showed that flooding time influenced the number of eggs laid and the development of eggs, especially in spring when the water flooded ahead of time was not conducive to the reproduction of *O. hupensis* [14]. The change of water level also affected the distribution height of the snail, which rose accordingly when the water level increased [15]. The change of water level also changed flow velocity of which affected the diffusion of *O. hupensis* [14]. However, after exploring the relationship between them in a long period, they found that there was not a simple linear relationship, but quadratic or cubic functions and more complex curve relations (with the change of these factors, there might be more than one range which was beneficial to *O. hupensis*) [16]. Soil temperature, humidity and vegetation height, coverage also affected *O. hupensis*. Generalized additive models was often used to analyze the relationship between *O. hupensis* density and various micro-environmental factors, and found that soil temperature and humidity content had U-shaped curve or more complex relationship with snail density [4, 11].

### Suitable ranges of micro-environmental factors for *O. hupensis* survival

Many researches also believed that there was a suitable range of vegetation height and coverage, which was conducive to the survival of *O. hupensis*. The range of flooding time, pH, soil temperature and humidity suitable for snail survival was 2–7 months, 5.5–7.9, 15–30 °C, and 20–80%, respectively [4, 11, 17–25]; vegetation coverage and height in weed community was 35–90%, 15–47 cm, *Carex* spp. and *Triarrhena* community (35–95%, 20–33 cm), single *Carex* spp. community (16–32%), single *Triarrhena lutarioriparia* community (80–157 cm), *Phragmites communis* community (1–100%, 72–78 cm), other herb communities (80–100%, 16–50 cm) [19, 22, 26, 27]. Only one article with the subspecies *O. h. quadrasi* was included in our study. The detailed references were also listed in Table 2.

**Table 2:** Suitable range of micro-environmental factors for *Oncomelania hupensis* survival and reproduction

Factors	Suitable range	Reference
Water level (flood duration)	4–5 months	[23]
	2–7 months	[24]
	24 m (3 months)–25 m (4 months)	[20]
	130–160 days	[18]
pH	6.6–7.0	[4]
	5.5–7.0	[11]
	5.6–7.9^a^	[17]
Soil temperature	16–19 °C	[22]
	16–20 °C	[21]
	23–24 °C	[4]
	24–29 °C	[11]
	15–30 °C	[19]
Soil humidity	0.2–0.3% (m^3^/m^3^)	[22]
	59–69%	[4]
	20–60%	[19]
	60–80%	[11]
	28–38%	[25]
Vegetation coverage	16–32% (*Carex* spp.)	[22]
	80–90% (herb community)	[26]
	60–100% (herb community)	[27]
	35–90% (weed)	[27]
	35–95% (*Carex* spp. and *Triarrhena lutarioriparia* community)	[27]
	1–100% (*Phragmites communis*)	[27]
Vegetation height	80–157 cm (*Triarrhena lutarioriparia*)	[22]
	16–20 cm (herb community)	[26]
	20–50 cm (herb community)	[27]
	15–47 cm (weed)	[27]
	20–33 cm (*Carex* spp. and *Triarrhena lutarioriparia* community)	[27]
	72–78 cm (*Phragmites communis*)	[27]

^a^*Oncomelania hupensis quadrasi*, the other subspecies were not specified

### Precise predictive potential of *O. hupensis* distribution using micro-environmental factors

Exploring the most suitable range for the survival of *O. hupensis* in different regions can be targeted to formulate measures to control snails, and it can also provide basic data for accurate prediction of the distribution of *O. hupensis*. In recent years, geographic information system (GIS) and remote sensing (RS) have been widely used in snail prediction. Using suitable growth conditions of *O. hupensis*, environmental factors were integrated into the prediction model to predict the potential habitats [28]. After combination with the intensity of different environmental factors, the region was divided into different risk levels [5]. Therefore, accurately identified environmental variables and appropriate range can help improve the accuracy of the prediction model and achieve the purpose of snail control.

## Micro-environmental factors with snail control potentials

Among all identified factors, snail control and schistosomiasis prevention forest, plant extracts, microorganisms, predators have been shown to have a negative impact on the snails. Micro-environmental factors that negatively affect *O. hupensis* can be used in schistosomiasis prevention and *O. hupensis* control.

### Plant extracts and microorganisms—potential molluscicides

Some plant extracts were found to have molluscicidal effects on *O. hupensis*. These extracts were phytochemicals or metabolites extracted from plants (Table 3). A total of 18 plant extracts were discovered, all of which had a damaging effect on the snail tissue in laboratory settings, mainly affect enzyme activity, interfere with energy metabolism, produce liver toxicity, and reduce the content of glycogen and protein [29–46]. The plant molluscicides were derived from extracts of flowers, fruits, roots, stems, and leaves of plants, and had the advantages of high efficiency, low toxicity, and easy degradation [47]. At present, researches on plant molluscicides are mostly focused on the screening and laboratory research of plants that can produce molluscicidal active substances. A plant molluscicide extracted from the seeds of *Camellia oleifera*, Luo-Wei, was developed in 2007. It had a good molluscicidal effect, but 4% Luo-Wei is moderately toxic to Japanese quail and shrimp, and highly toxic to zebra fish [48].

**Table 3:** Plant extracts with molluscicidal effects on *Oncomelania hupensis*

Botanic scientific name	Extracts	Reference
*Nerium indicum*	Triterpenoid saponins	[43]
*Arisaema heterophyllum*	Calcium oxalate	[36]
*Ginkgo biloba*	Ethanolic extracts	[39]
*Eomecon chionantha*	Alkaloid	[35]
*Solanum xanthocarpum*	Unspecified extracts	[46]
*Cinnamomum camphora*	Leaf extracts	[32]
*Macleaya cordata*	Benzo[c]phenanthridine alkaloids	[34]
*Dioscorea zingiberensis*	Saponins	[42]
*Reineckia carnea*	Unspecified extracts	[44]
*Phytolacca americana*	Leaf extracts	[33]
*Melia azedarach*	Leaf extracts	[31]
*Buddleja lindleyana*	Active ingredient (AIBL)	[30]
*Sapium sebiferum*	Ethanolic extracts	[38]
*Alternanthera philoxeroides*	Aqueous extract	[41]
*Euphorbia fischeriana*	Ethanolic extracts	[37]
*Glycyrrhiza uralensis*	*Glycyrrhiza uralensis* extracts	[29]
*Rumex japonicus*	Unspecified extracts	[45]
*Camptotheca acuminata*	Camptothecin	[40]

AIBL: Active ingredient of *Buddleja lindleyana*

Besides, some microorganisms isolated from the soil, water, plants and snails itself had similar effects [49]. Through *O. hupensis* snail’s toxicity experiments, *Aspergillus fumigatus* SL-30, *Streptomyces violaceoruber*, *Xanthobacter autotrophicus*, strain JJ18 from the endophytic fungi of *Pseudolarix amabilis.* were proven to have good molluscicidal effects in laboratory settings [50–55]. Results of high-throughput sequencing technology, showed that the abundance, diversity and distribution uniformity of bacterial community in snail gathering areas were better than those in the non-snail breeding areas [56]. These suggest that microorganisms extracted from plants and soil can be used to develop potential molluscicides. These newly emerged microbial molluscicides were efficient and environmental friendly [57]. Researches on plant and microbial molluscicides were mostly at laboratory stage. Further field application tests and product development are not on the agenda. One of the reason could be that the molluscicidal mechanism is still not clear, safety issues on animal or human beings are not addressed and industrializing techniques are not sufficient [49]. Another reason may be that niclosamide is the only snail killer recommended by WHO since 1972 [58], and has a good snail killing effect [59], so the development of new drugs has not been given priority. However, despite of different dosage forms developed to expand its application scope [60], niclosamide remained highly toxic to fish. In this case, we suggest strengthening the study and development of plant-extracting and microbial molluscicides and related technologies, and evaluating their molluscicidal effect and environmental friendliness.

### Forestry schistosomiasis control project—mechanism, achievements and challenges

China launched forestry schistosomiasis control project in 2006, and planted a total of 5.189 billion m^2^ of *O. hupensis* control and schistosomiasis prevention forest in 10 years [61]. On one hand, the programs were designed to control snails by creating forests to change *O. hupensis* breeding environment [62]. It was found enzyme levels in *O. hupensis* in the environments of the forestry schistosomiasis control project were different from those in the snail natural habitats. This suggested that snail enzyme and energy metabolism might be interfered by the forests [63]. On the other hand, the snail-inhibiting active ingredients produced and released by plants showed an allelopathic effect on *O. hupensis* to achieve biological snail-inhibition [64]. Liquors of *Liquidambar formosana*, *Sapium sebiferum* and *Pterocarya stenoptera* had strong allelopathy to *O. hupensis* [65]. This measure has achieved remarkable results. Since the start of this program, the density of snails had decreased by 89.9%, and the density of infected snails had decreased by 95.8% in 10 years [61]. In addition, the project also increased the forest coverage, and played a positive role in water conservation and soil erosion control as rainwater could be intercepted by tree canopy and soils could be fixed by tree roots [66]. However, afforestation based on a single plant genus (*Populus*) significantly reduced the original vegetation diversity of the beach [67]. Therefore, different models of forestry projects were needed. The density of *O. hupensis* was different among various plant species. Snails were less distributed in *Cynodon dactylon*, *Alternanthera philoxeroides*, *Pterocarya stenoptera* community and *Nerium indicum* community [25, 68, 69]. *Salix babylonica*, *Liquidambar formosana*, *Taxodium* hybrid ‘zhongshanshan’, *Taxodium ascendens*, had better adaptability in marshland and lakeside land, and could be used for forestry project [70]. Agroforestry snail control forests, such as “*Juglans regia* + *Allium sativum*”, “*Juglans regia* + *Capsicum annuum*” and other intercropping patterns, were proved to have good molluscicidal effect and good economic value [71]. In recent years, the forestry schistosomiasis control project faces many challenges. As artificial afforestation will have a certain impact on wetlands ecology, it may have potential conflict with the current trend of environmental protection policies in China. It will be the future direction to design new environmentally friendly forestry schistosomiasis control project with ecological diversity.

### Predator

*Procambarus clarkia *(also known as crayfish) and *Ophiocephalus argus* (black carp) competed with *O. hupensis* for food and ecological space, and were at the upper end of the food chain, which could effectively control and kill *O. hupensis* [72–74]. Experimental studies and semi-field evaluations also demonstrated that crayfish could effectively reduce the population of *O. hupensis* through predatory interactions [75]. However, the complexity of habitat could strongly affect the intensity of predation in natural communities. In the large-scale wild habitats, the environment was complex, and many different micro-environments could provide suitable refuges for the survival of the snails, which increased the difficulty of predation, especially the predation efficiency of the smaller snails [76]. There was still a lot of uncertainty when this method was used to control snails. Therefore, a long-term field investigation is needed to evaluate the effectiveness and feasibility of this ecological snail control method.

## Micro-environmental factors with further researches needed

The effect of micro-environmental factors like water quality and soil indexes on *O. hupensis* was also reported. Snails were more likely to survive in water with high transparency [77]. By comparing the differences between the chemical indexes of the water bodies of snail-breeding beach and natural extinction beaches, it was found that the high pH and the fluorine ion (F^−^) content in the water body may be related to the natural extinction of *O. hupensis* [78]. Field investigation in Fu River, China, also showed that the significant increasing in natural mortality of *O. hupensis* might be related to potential water pollution [79]. On the contrary, another field survey [80] showed that exceeding the standard of nitrogen and phosphorus in Dongting Lake, China, leading to eutrophication, was conducive to the survival of *O. hupensis*. The survival experiments of *O. hupensis* in environments with different water quality also indicated that water quality may affect *O. hupensis*, but further evidences were needed [81, 82]. Similarly, studies have shown that there was a correlation between soil fertility indexes (organic compounds, total N, total P, total K, etc.) and snail density, but there was no consistent conclusion [83–85].

Compared to the variety of water/soil physical and chemical properties, the studies focus on their impact to *O. hupensis* were very few. It could be due to the following reasons: (i) There are numerous physical and chemical indicators and components in the water and soil environment. Different types and degrees of pollution have different effects on snails. More well-designed studies are required to reflect the impact of various indicators on *O. hupensis*; (ii) Short-term field investigations or experiments may not obtain solid evidence of the impact of water quality and soil components on snails, which could be a long-term process; (iii) Long-term retrospective analysis requires the support of historical water quality data with cooperation between multiple departments. Therefore, more targeted researches are needed, and if necessary, it is better to cooperate different environmental factors in the study design. The monitoring of water quality changes can help us to identify potential snail habitats, improve the snail monitoring and early warning system, and provide theoretical basis for existing and new efficient snail control methods. The clear identification of the impacts of environmental factors that can help with the effective reduction of the snail density.

There are nine subspecies of *O. hupensis*, which are from different environments in East and South East Asia. For instance, *O. h. hupensis* are from marshland areas near Yangtze River in China, *O. h. quadrasi* are mainly from the islands in the Philippines while *O. h. robertsoni* from mountainous areas prefers elevated areas [86]. The role of various types of micro-environments playing in snail survival may be different and it could contribute in the design of subspecies-specific snail control approach. For example, snail subspecies that prefers marshland and are closely connected with agricultural activities, like *O. h. hupensis* and *O. h. nosophora* could be efficiently controlled with the environmental transformation methods including ditch lining; while for snails living in hilly areas, such as *O. h. robertsoni* and *O. h. h.* fausti strain, flood storage could be an optimal choice [86, 87]. However, most of the literatures included did not specify the subspecies of *O. hupensis*. More information on different subspecies deserves to be studied in future research.

Most of the included studies were about the relevant impact on *O. hupensis* survival, very few covered the impact on its growth and reproduction, which is also essential for the snail population. Possible reason could be the difficulty of laboratory study on *O. hupensis* reproduction. But this kind of study is equally important for snail control strategy and requires more researches in the future.

## Conclusions

This scoping review found many micro-environmental factors including water level, pH value, soil temperature, soil humidity, the coverage and height of vegetation could affect *O. hupensis*. Successful use of these factors could benefit the surveillance and control of snail habitats. Water quality and soil composition as well as the technology and application of bio-molluscicides which could be more environmentally friendly deserve more attention. We call for further comprehensive studies to improve the accuracy of snail prediction, provide a better theoretical basis for its effective control, and inspire novel control ideas.

## Supplementary Information

**Additional file 1.** Search strategy by database.

## Data Availability

The datasets used and/or analyzed during the current study are available from the corresponding author on reasonable request.

## Abbreviations

GIS: Geographic information system
RS: Remote sensing
