# Supplementary material for: Impact of micro-environmental factors on survival, reproduction and distribution of Oncomelania hupensis snails
Source: Infect Dis Poverty. 2021 Apr 7;10:47. doi: 10.1186/s40249-021-00826-3 (PMC8028213; doi:10.1186/s40249-021-00826-3)
Supplement: Supplementary file 1 — Additional file 1. Search strategy by database. [file 40249_2021_826_MOESM1_ESM.docx]

**Additional file 1 search strategy by database**

**1、key concepts**

| Micro-environment | Oncomelania |
| --- | --- |

**2、Associated keywords:**

| **Micro-environment** | Oncomelania |
| --- | --- |
| Water quality | Snail* |
| Microorganism/germ | *O. hupensis quadrasi* |
| Predator | *O. hupensis nosophora* |
| aquatic vegetation | *O. hupensis lindoensis* |
| Water temperature | *O. hupensis tangi* |
| Water depth | *O. hupensis formosana* |
| water turbidity | *O. hupensis chiui* |
| Nitrogen | *O. hupensis robertsoni* |
| Phosphate/phosphorus | *Oncomelania minima* |
| Sulfate |  |
| Chloride*/muriate |  |
| PH |  |
| DO（dissolved oxygen) |  |
| COD(chemical oxygen demand) |  |
| BOD(biochemical oxygen demand) |  |
| TOC  (total organic carbon) |  |
| TOD  (total oxygen demand) |  |
| Heavy metal /lead/Pb/mercury/Hg/arsenic/As/cadmium/Cd/chromium/Cr/Cu/copper/Fe |  |
| K(potassium) |  |
| Na(sodium) |  |
| Ca(calcium) |  |
| Mg(magnesium) |  |
| F（fluorine） |  |
| Soil |  |
| Vegetation |  |
| Eco* |  |

And use these words (Effect* or Influence or Correlat* or Related or Relevance or Association or Relation* or growth or Propagat* or Reproduc*or Breed* or development) to limit the results.

**3、Databases to search:**

Scientific literature: PubMed, EBSCOhost, Institute for Scientific Information Web of science, China National Knowledge Infrastructure (CNKI), and Chinses scientific journal database wan fang)

**Pubmed**

(soil [Title/Abstract] or vegetation[Title/Abstract] or water quality[Title/Abstract] or ph[Title/Abstract] or aquatic vegetation[Title/Abstract] or “dissolved

oxygen”[Title/Abstract] or “chemical oxygen demand”[Title/Abstract] or “biochemical oxygen demand”[Title/Abstract] or microorganism[Title/Abstract] or germ[Title/Abstract] or Chloride[Title/Abstract] or Heavy metal[Title/Abstract] or “total organic carbon”[Title/Abstract] or “total oxygen demand”[Title/Abstract] or sulfate[Title/Abstract] lead[Title/Abstract] or Pb[Title/Abstract] or mercury[Title/Abstract] or Hg[Title/Abstract] or arsenic[Title/Abstract] or As[Title/Abstract] or cadmium[Title/Abstract] or Cd[Title/Abstract] or chromium[Title/Abstract] or Cr[Title/Abstract] or Cu[Title/Abstract] or copper[Title/Abstract] or Fe[Title/Abstract] or K[Title/Abstract] or potassium[Title/Abstract] or Na[Title/Abstract] or sodium[Title/Abstract] or Ca[Title/Abstract] or calcium[Title/Abstract] or Mg[Title/Abstract] or magnesium[Title/Abstract] or F[Title/Abstract] or fluorine[Title/Abstract] or Water temperature[Title/Abstract] or Water depth[Title/Abstract] or water level[Title/Abstract] or hardness[Title/Abstract] or water turbidity[Title/Abstract] or nitrogen[Title/Abstract] or Phosphorus/[Title/Abstract] or Phosphate[Title/Abstract] or ecolog*[Title/Abstract] or predator*[Title/Abstract]) and ((snail*[Title/Abstract] AND (japonicum[Title/Abstract] )) or oncomelania or O. hupensis quadrasi or O. hupensis nosophora or O. hupensis lindoensis or O. hupensis tangi or O. hupensis formosana or O. hupensis chiui or O. hupensis robertsonior Oncomelania minima) and (Effect*[Title/Abstract] or Influence[Title/Abstract] or Correlat*[Title/Abstract] or Related[Title/Abstract] or Relevance[Title/Abstract] or Association[Title/Abstract] or Relation*[Title/Abstract] or Growth[Title/Abstract] or Propagat*[Title/Abstract] or Reproduc*[Title/Abstract] or Breed*[Title/Abstract] or development[Title/Abstract] )

**Web of science**

TS=(soil or vegetation or water quality or ph or aquatic vegetation or dissolved oxygen or chemical oxygen demand or biochemical oxygen demand or microorganism or germ or Chloride or Heavy metal or total organic carbon or total oxygen demand or sulfate or lead or Pb or mercury or Hg or arsenic or As or cadmium or Cd or chromium or Cr or Cu or copper or Fe or K or potassium or Na or sodium or Ca or calcium or Mg or magnesium or F or fluorine or Water temperature or Water depth or water level or hardness or water turbidity or nitrogen or Phosphorus or Phosphate or encolog* or predator* ) and TS=((Snail* and (japonicum)) or oncomelania or O. hupensis quadrasi or O. hupensis nosophora or O. hupensis lindoensis or O. hupensis tangi or O. hupensis formosana or O. hupensis chiui or O. hupensis robertsonior Oncomelania minima) and TS=(Effect* or Influence or Correlat* or Related or Relevance or Association or Relation* or Growth or Propagat* or Reproduc* or Breed* or development)

**EBSCOhost**

SU(soil or vegetation or water quality or ph or aquatic vegetation or dissolved oxygen or chemical oxygen demand or biochemical oxygen demand or microorganism or germ or Chloride or Heavy metal or total organic carbon or total oxygen demand or sulfate or lead or Pb or mercury or Hg or arsenic or As or cadmium or Cd or chromium or Cr or Cu or copper or Fe or K or potassium or Na or sodium or Ca or calcium or Mg or magnesium or F or fluorine or Water temperature or Water depth or water level or hardness or water turbidity or nitrogen or Phosphorus or Phosphate or ecolog* or predator* ) and SU((Snail* and (japonicum)) or oncomelania or O. hupensis quadrasi or O. hupensis nosophora or O. hupensis lindoensis or O. hupensis tangi or O. hupensis formosana or O. hupensis chiui or O. hupensis robertsonior Oncomelania minima) and SU(Effect* or Influence or Correlat* or Related or Relevance or Association or Relation* or Growth or Propagat* or Reproduc* or Breed* or development)

**CNKI**

SU=(土壤+植被+水质+微生物+水生植物+水温+水深+浑浊度+水位+硬度+氮+磷+硫酸盐+氯化物+Ph +溶解氧+化学耗氧量+生化需氧量+总有机碳+总需氧量+重金属+铅+Pb+汞+Hg+砷+镉+Cd+铬+Cr+铜+铁+钾+钠+钙+镁+氟+捕食者+生态环境) * 钉螺 *(影响+相关+关联+关系+生长+发育+繁殖+生存)

**Wan Fang**

主题:(土壤+植被+水质+微生物+水生植物+水温+水深+水位+硬度+浑浊度+氮+磷+硫酸盐+氯化物+Ph +溶解氧+"化学耗氧量"+"生化需氧量"+"总有机碳"+"总需氧量"+重金属+铅+Pb+汞+Hg+砷+镉+Cd+铬+Cr+铜+铁+钾+钠+钙+镁+氟+捕食者+生态环境) and 主题: 钉螺 and 主题：(影响+相关+关联+关系+生长+发育+繁殖+生存)

**4、search results**

| Database | Number of documents found |
| --- | --- |
| pubmed | 123 |
| Web of science | 240 |
| EBSCOhost | 49 |
| CNKI | 228 |
| Wanfang | 619 |
| Total | 1259 |

Records after duplicates removed : 1034
